# Evaluating lower limb tendinopathy with Victorian Institute of Sport Assessment (VISA) questionnaires: a systematic review shows very-low-quality evidence for their content and structural validity—part I

**DOI:** 10.1007/s00167-021-06598-5

**Published:** 2021-05-21

**Authors:** Vasileios Korakakis, Argyro Kotsifaki, Manos Stefanakis, Yiannis Sotiralis, Rod Whiteley, Kristian Thorborg

**Affiliations:** 1 Aspetar, Orthopaedic and Sports Medicine Hospital, PO 29222, Doha, Qatar; 2 Hellenic Orthopaedic Manipulative Therapy Diploma (HOMTD), Athens, Greece; 3 School of Science, Program of Physiotherapy, University of Nicosia, Nicosia, Cyprus; 4 Department of Orthopaedic Surgery, Sports Orthopedic Research Center-Copenhagen (SORC-C), Amager-Hvidovre Hospital, Faculty of Health Sciences, Copenhagen University, Copenhagen, Denmark

**Keywords:** Patient-reported outcome measures, Tendinopathy, Content validity, Unidimensionality, COSMIN

## Abstract

**Purpose:**

The Victorian Institute of Sport Assessment (Achilles tendon—VISA-A, greater trochanteric pain syndrome—VISA-G, proximal hamstring tendinopathy—VISA-H, patellar tendon—VISA-P) questionnaires are widely used in research and clinical practice; however, no systematic reviews have formally evaluated their content, structural, and cross-cultural validity evidence. The measurement properties referring to content, structural and cross-cultural validity of the VISA questionnaires were appraised and synthesized.

**Methods:**

The systematic review was conducted according to Consensus-based Standards for the Selection of Health Measurement Instruments (COSMIN) methodology. PubMed, Cochrane, CINAHL, EMBASE, Web of Science, SportsDiscus, grey literature, and reference lists were searched. Development studies and cross-cultural adaptations (12 languages) assessing content or structural validity of the VISA questionnaires were included and two reviewers assessed their methodological quality. Evidence for content (relevance, comprehensiveness, and comprehensibility), structural, and cross-cultural validity was synthesized. A modified Grading of Recommendations Assessment Development and Evaluation (GRADE) approach was applied to evidence synthesis.

**Results:**

The VISA-A presented very-low-quality evidence of sufficient relevance, insufficient comprehensiveness, and inconsistent comprehensibility. VISA-G displayed moderate-quality evidence for sufficient comprehensibility and very-low-quality evidence of sufficient relevance and comprehensiveness. The VISA-P presented very-low-quality evidence of sufficient relevance, insufficient comprehensiveness, and inconsistent comprehensibility, while VISA-H presented very-low evidence of insufficient content validity. VISA-A displayed low-quality evidence for structural validity concerning unidimensionality and internal structure, while VISA-H presented low-quality evidence of insufficient unidimensionality. The structural validity of VISA-G and VISA-P were indeterminate and inconsistent, respectively. Internal consistency for VISA-G, VISA-H, and VISA-P was indeterminate. No studies evaluated cross-cultural validity, while measurement invariance across sexes was assessed in one study.

**Conclusions:**

Only very-low-quality evidence exists for the content and structural validity of VISA questionnaires when assessing the severity of symptoms and disability in patients with lower limb tendinopathies.

**Level of evidence:**

IV.

**Registration:**

PROSPERO reference—CRD42019126595.

**Supplementary Information:**

The online version contains supplementary material available at 10.1007/s00167-021-06598-5.

## Introduction

According to the International Scientific Tendinopathy Symposium Consensus from 2020, the impact of lower limb tendinopathies on the patient should be measured using validated outcome measures that can capture the core domains of the condition such as: functional testing, participation in life activities, psychological factors, physical function capacity, and most importantly disability via condition-specific patient-rated outcome measures (PROMs) [44, 78]. The Victorian Institute of Sport Assessment (VISA) questionnaires [6, 20, 69, 80] have been recommended by the 2020 consensus statement [78] and are the most used condition-specific lower limb questionnaires in the literature [8, 11, 37, 44, 52, 57, 76, 77].

Four self-administered VISA questionnaires exist which assess the severity of symptoms in patients with Achilles tendinopathy (VISA-A), greater trochanteric pain syndrome (VISA-G), proximal hamstring tendinopathy (VISA-H), and patellar tendinopathy (VISA-P) [6, 20, 69, 80]. Six out of eight items rate pain level during daily activities and functional tests, and two items provide information on the impact of tendinopathy in physical activity or sports participation.

The strength of a PROM can be evaluated by the COnsensus-based Standards for the selection of health Measurement INstruments (COSMIN) [64]. COSMIN evaluates validity, reliability, and responsiveness of outcome measurement instruments like the patient-reported VISA questionnaires [6, 20, 69, 80]. The quality of an outcome measurement instrument is determined by its validity [55]. In turn, content validity has been suggested as the first and most important measurement property to consider when selecting a PROM [65, 74]. Lack of content validity potentially affects all other measurement properties. For example, irrelevant items decrease internal consistency and structural validity, and missing concepts decrease validity and responsiveness [74].

Despite the widespread use of the VISA questionnaires, to our knowledge, no systematic reviews have formally evaluated their content, structural, and cross-cultural validity evidence.

The measurement properties of the VISA questionnaires were appraised and synthesized. Here, the first part of the systematic review is reported of all available VISA questionnaires for patients with Achilles tendinopathy, greater trochanteric pain syndrome, proximal hamstring tendinopathy, and patellar tendinopathy, providing researchers and clinicians with an overview appraising measurement properties concerning content, structural, and cross-cultural validity using COSMIN methodology.

## Materials and methods

### Protocol and registration

The search strategy and reporting of this systematic review adhered to the Preferred Reporting Items for Systematic Reviews and Meta-Analyses (PRISMA) guidelines [53], followed the COSMIN methodology for systematic review of PROMs [64], and the Cochrane group’s recommendations [29]. The protocol was prospectively registered in PROSPERO (CRD42019126595).

### Information sources and search methods

PubMed, Cochrane, CINAHL, EMBASE, Web of Science, and SportsDiscus databases were independently searched by two reviewers from inception of database to 19 May 2020 without language restriction, to reduce language and publication bias.

Grey literature was searched via OpenGrey.eu, and the following registries: Clinical Trials.gov and EU clinical trials register. Reference lists, citation tracking results, and systematic reviews were also manually searched.

The search strategy included a comprehensive PROM filter developed by the COSMIN group [13, 73] and two basic strings of key terms (names of instruments and population of interest) (Online Resource 1).

### Study selection

Search results were imported into EndNote where duplicates were removed, and then, title and abstract were independently evaluated by two reviewers (AK and MS). Subsequently, the full text for each potentially eligible study was evaluated. Reference lists were checked for additional potentially relevant studies [64]. A third reviewer (VK) was consulted if consensus was not reached [39].

This part of the systematic review included only the eligible studies that reported on content validity, structural validity, internal consistency, and cross-cultural validity/measurement invariance of the VISA questionnaires. The remaining measurement properties are reported and synthesized separately.

### Eligibility criteria

Content validity studies were eligible if they were full-text original articles assessing relevance, comprehensibility, or comprehensiveness of the content of the VISA questionnaires by patients or professionals. Cross-cultural adaptation studies of the questionnaires were included as content validity studies if they performed a pretest of the adapted VISA [2, 10]. Studies evaluating the internal structure of the questionnaires (structural validity, internal consistency, and cross-cultural validity/measurement invariance) were eligible if they were full-text original articles assessing the dimensionality of the construct of the questionnaires by factor analysis, reporting on the interrelatedness among the items (Cronbach’s *α*), or evaluating if the performance of the items on a culturally adapted VISA were an adequate reflection of the performance of the items of the original version of the instrument [13, 64].

### Inclusion and exclusion criteria

The general inclusion criteria were: (a) all types of studies assessing at least one measurement property of the VISA questionnaires (including development and not limited to validity, reliability, responsiveness, and interpretability), (b) including patients with greater trochanteric pain syndrome, proximal hamstring tendinopathy, patellar tendinopathy, or Achilles tendinopathy, as well as other groups of asymptomatic/injured individuals that were used in measurement properties assessment, and (c) only full-text articles in peer-reviewed journals. Following recommendations [64], studies that only used a VISA questionnaire as an outcome measurement instrument were excluded, for instance randomized-controlled trials, or studies in which a VISA was used in a validation study of another instrument.

### Data extraction

Data from studies meeting the inclusion criteria were extracted independently (VK and AK) using standardized extraction forms and cross-checked. Any disagreements were resolved by consensus. Publication details, sample size, patient characteristics, content validity domain evaluated and population (relevance, comprehensiveness, comprehensibility), analysis and model of structural validity assessment, and main indices and results for structural validity and internal consistency (i.e., number of factors, Cronbach’s *α*) were extracted.

### Assessment of the methodological quality of single studies and evaluation of results against criteria for good measurement properties

The methodological quality of each eligible study on a measurement property was assessed separately using the COSMIN Risk of Bias checklist [54]. The development studies and the studies on content validity were assessed using COSMIN standards; 35 items subdivided into two parts and 31 items subdivided into two parts (patients or professionals), respectively. Studies assessing internal structure were also evaluated against COSMIN standards; 4 items for structural validity, 5 items for internal consistency, and 4 items for cross-cultural validity or measurement invariance. COSMIN recommendations [17, 21] were used to judge important flaws in structural validity. Measurement properties from first administration of each PROM were used for evaluation where applicable.

Each standard and subsequently each study was scored on a 4-point rating scale as “very good”, “adequate”, “doubtful”, or “inadequate” [64]. The methodological study quality score per measurement property was determined by the item with the lowest score (worse score counts) [64].

Subsequently, the results on each measurement property were rated against the updated criteria for good measurement properties [64, 72]. Content validity and internal structure were rated as “sufficient” (+), “insufficient” (−), “inconsistent” (±), or “indeterminate” (?). Additional criteria for structural validity and internal consistency good measurement properties were applied [10]. Two reviewers (AK and MS) independently rated the quality of measurement properties; in case of any rating discrepancies, consensus was resolved by discussion with a third reviewer (VK).

### Rating the quality of evidence

The evidence was summarized, and the quality of evidence was judged for each measurement property separately by two independent reviewers (AK and MS) using a modified GRADE approach [64]. Evidence started at high quality and was downgraded according to the presence and extent of specific dimensions recommended for the quality of evidence in PROM measurement properties studies: risk of bias (methodological quality), inconsistency (unexplained inconsistency of results across studies), imprecision (total sample size), and indirectness (evidence from population different than that of interest). For content validity, only risk of bias, inconsistency, and indirectness are applicable [74]. The results were qualitatively summarized or quantitatively pooled (where applicable) and compared against the criteria for good measurement properties to determine whether the “overall” measurement property of the PROM is sufficient (+), insufficient (−), inconsistent (±), or indeterminate (?) [64].

### Statistical analysis

Pooling of internal consistency coefficients was performed using the R statistical platform [66] (metafor package) [79]. Initially, the coefficients were transformed [24] to stabilize the variances and approximate to the normal distribution. A random-effects model was used due to clinical and statistical heterogeneity and subgroup analyses were performed based on clinical criteria (i.e., patients or mixed sample of patients and asymptomatic individuals, age) where applicable. Values were presented as pooled mean estimate and 95% confidence intervals (95% CI).

## Results

### Study characteristics

**Fig. 1:**
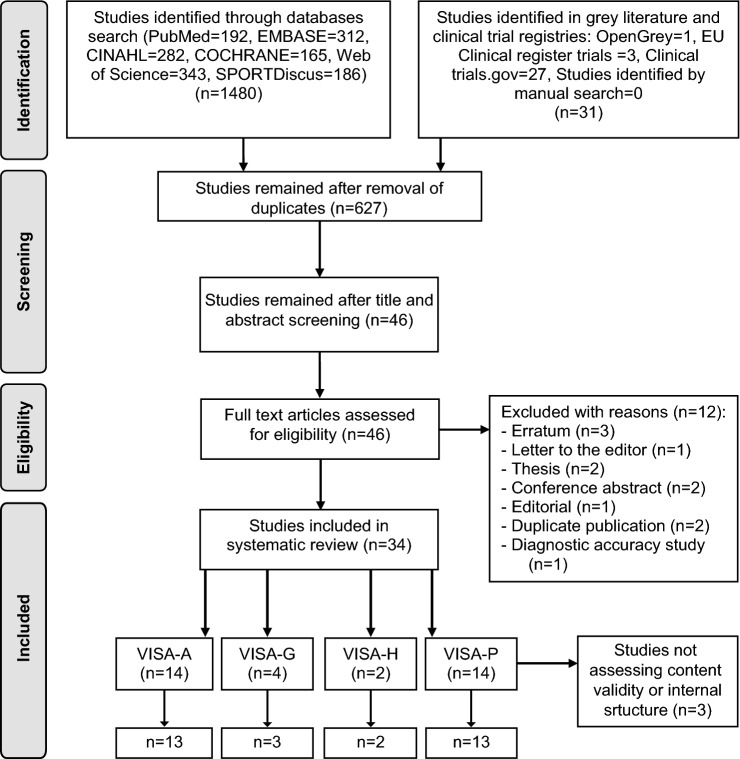
PRISMA flow diagram for study inclusion

Of the original 1511 studies, 34 remained after duplicate removal. Of these, 31 met the eligibility criteria appraising measurement properties of: VISA-A [15, 16, 18, 28, 30, 33, 34, 41, 43, 45, 51, 69–71], VISA-G [3, 19, 20, 31], VISA-H [6, 40], and VISA-P [1, 7, 22, 25–27, 32, 36, 42, 46, 60, 80, 81, 83]. Twenty-four cross-cultural adaptations validated the VISA questionnaires in 12 languages. Of the eligible studies, three [19, 27, 51] did not evaluate content validity and/or internal structure of the PROMs and were excluded (Fig. 1).

### Quality, results, and evidence synthesis of content validity studies

Content validity of the VISA questionnaires was evaluated by 71 patients (comprehensibility) with tendinopathy and 12 professionals (relevance). The VISA development studies were of inadequate quality [6, 20, 69, 80]. The quality of the VISA content validity studies is presented in Table 1, and the quality of evidence is presented in Table 2.

**Table 1:** COSMIN quality evaluation of the VISA content validity studies

Study	PROM	Asking patients			Asking experts	
		Relevance	Comprehensiveness	Comprehensibility	Relevance	Comprehensiveness
Silbernagel et al. [71]	VISA-A			D		
Dogramaci et al. [18]	VISA-A	D		D		
Kaux et al. [33]	VISA-A			D		
De Mesquita et al. [16]	VISA-A				D	
Hernandez-Sanchez et al. [28]	VISA-A			D		
Beaudart et al. [3]	VISA-G			D		
Jorgensen et al. [31]	VISA-H			D		
Locquet et al. [40]	VISA-H			D		
Hernandez-Sanchez et al. [26]	VISA-P			D		
Lohrer et al. [42]	VISA-P				I	
Korakakis et al. [36]	VISA-P				D	
Acharya et al. [1]	VISA-P			D		

*A* Achilles, *D* doubtful, *G* greater trochanteric pain syndrome, *H* hamstring, *I* inadequate

**Table 2:** Evidence synthesis on the content and structural validity of Victorian Institute of Sport Assessment questionnaires

PROM	Content validity							Structural validity		
	Relevance			Comprehensiveness		Comprehensibility				
	Study references	Rating of results	Quality of evidence	Rating of results	Quality of evidence	Rating of results	Quality of evidence	Study references	Rating of results	Quality of evidence
VISA-A	[16, 18, 28, 33, 71]	+	Very low^a^	–	Very low^a^	±	Very low^a^	[28, 71]	+	Low^d,e^
VISA-G	[3, 20, 31]	+	Very low^a^	+	Very low^a^	+	Moderate	[20]	?	?^b^
VISA-H	[6, 40]	–	Very low^a^	–	Very low^a^	–	Very low^a^	[6]	–	Low^c,d^
VISA-P	[1, 26, 36, 42, 80]	+	Very low^a^	–	Very low^a^	±	Very low^a^	[7, 22, 25, 26, 36, 83]	±	NR^f^

*NR* not rated, *PROMs* patient-reported outcome measures, *(* +*)* sufficient results, *(–)* insufficient results, *(* ±*)* inconsistent results, *(?)* indeterminate results

^a^Based only on reviewers’ ratings

^b^Indeterminate evidence due to lack of sufficient detail for VISA-G structure

^c^Risk of bias (serious): only one study of adequate quality

^d^Indirectness: only part of the study population were patients

^e^Inconsistency: inconsistent structural validity results between studies

^f^Structural validity study results were inconsistent, and according to COSMIN guidelines, the evidence cannot be graded

#### VISA-A

Very-low-quality evidence was found for inconsistent content validity of VISA-A (Table 2).

#### VISA-G

Very-low-quality evidence for sufficient content validity of VISA-G (Table 2).

#### VISA-H

Very low quality of evidence was found for VISA-H (Table 2).

#### VISA-P

Four cross-cultural adaptations [25, 27, 46, 81] did not evaluate content validity. Very-low-quality evidence was found for inconsistent content validity of VISA-P (Table 2).

### Quality, results, and evidence synthesis of studies evaluating structural validity and internal consistency

#### VISA-A

Low-quality evidence indicated sufficient unidimensionality for VISA-A (Tables 2, 3).

**Table 3:** Quality assessment and results of the structural validity and internal consistency of VISA questionnaire studies

VISA-A	Country (language)	Structural validity				Internal consistency			
		*n*	COSMIN quality rating	Analysis (model)	Result (rating)	*n*	COSMIN quality rating	Result (rating)	Comments
Silbernagel et al. [71]	Sweden (Swedish)	51	Doubtful	PCA with VR on factors with eigenvalues > 1	Two factors, but explained variance was not reported (?)	51	Inadequate	Cronbach *α* = 0.77 (+)	Evaluated in patients
de Knikker et al. [15]	Netherlands (Dutch)	NT				17	Very good	Cronbach *α* = 0.58 (items 1–8) (−)	Evaluated in patients Items 1–3 *α* = 0.79 Items 4–6 *α* = 0.72 Items 7–8 *α* = 0.57
Maffulli et al. [45]	Italy (Italian)	NT				50	Inadequate	Cohen’s *k* = 0.80	Inadequate internal consistency statistic
Lohrer et al. [41]	Germany (German)	NT				30	Very good	Cronbach *α* = 0.74 (+)	AT patients (*n* = 15) and pre-op AT (*n* = 15)
Lohrer et al. [43]	Germany (German)	NT				39	Doubtful	Cronbach *α* = 0.87 (+)	Recruited patients with Haglund’s disease
Dogramaci et al. [18]	Turkey (Turkish)	NT				55	Very good	Cronbach *α* = 0.66 (−)	Evaluated in patients
Iversen et al. [30]	Denmark (Danish)	NT				71	Very good	Cronbach *α* = 0.73 (+)	Evaluated in patients
Kaux et al. [33]	Belgium (French)	NT				116	Very good	Cronbach *α* = 0.90 (+)	Evaluated in both patients and asymptomatic
Hernandez-Sanchez et al. [28]	Spain (Spanish)	210	Very good	CFA	1-factor solution tested *X*^2^ = 47.03 (*p* < 0.01), SRMR = 0.03, CFI 0.98, RMSEA = 0.08 (+)	210	Very good	Cronbach *α* = 0.89 (+)	Evaluated in both patients and asymptomatic
De Mesquita et al. [16]	Brazil (Brazilian Portuguese)	NT				106	Very good	Cronbach *α* = 0.79 (+)	Evaluated in both patients and asymptomatic
Sierevelt et al. [70]	Netherland (Dutch)	NT				52	Very good	Cronbach *α* = 0.78 (athletes and non-athletes) (+)	Athletes (*n* = 39) *α* = 0.72 Non-athletes (*n* = 13) *α* = 0.82
Pooled or summary result (overall rating)		261	1-factor structure—sufficient (+)			708	> 75%—sufficient (+)		

VISA-G	Country (language)	Structural validity				Internal consistency			
		*n*	COSMIN quality rating	Analysis (model)	Result (rating)	*n*	COSMIN quality rating	Result (rating)	Comments
Fearon et al. [20]	Australia (English)	83	Doubtful	PCA	Explained variance was not reported (?)	83	Doubtful	Cronbach *α* = 0.52 (0.29–0.71) (?)	Criteria for “at least low evidence for sufficient structural validity” not met
Beaudart et al. [3]	Belgium, France (French)	NT				106	Doubtful	Cronbach *α* = 0.81 (?)	Criteria for “at least low evidence for sufficient structural validity” not met
Jorgensen et al. [31]	Denmark (Danish)	NT				49	Doubtful	Cronbach *α* = 0.98 (?)	Criteria for “at least low evidence for sufficient structural validity” not met
Pooled or summary result (overall rating)		83	Structure—indeterminate (?)			296	Internal consistency—indeterminate (?)		

VISA-H	Country (language)	Structural validity				Internal consistency			
		*n*	COSMIN quality rating	Analysis (model)	Result (rating)	*n*	COSMIN quality rating	Result (rating)	Comments
Cacchio et al. [6]	Italy (English)	60	Adequate	PCA with VR on factors with eigenvalues > 1 73.4%	Two factors accounted for 34.1% and 39.3% of variance, respectively (−)	60	Inadequate	Cronbach *α* = 0.84 (0.77–0.89)	Ignored due to evidence that VISA-H is not unidimensional
Locquet et al. [40]	Belgium (French)	NT				16	Inadequate	Cronbach *α* = 0.849	Ignored due to evidence that VISA-H is not unidimensional
Pooled or summary result (overall rating)		60	2-factor structure—insufficient (−)			76	Non-applicable		

VISA-P	Country (language)	Structural validity				Internal consistency			
		*n*	COSMIN quality rating	Analysis (model)	Result (rating)	*n*	COSMIN quality rating	Result (rating)	Comments
Frohm et al. [22]	Sweden (Swedish)	51	Adequate	PCA with VR on factors with eigenvalues > 1 85.0%	Three factors accounted together for 85% of the variance (First: items 2–6, & 8; Second: item 1; Third: item 7) (?)	51	Doubtful	Cronbach *α* = 0.83 (?)	Evidence that scale was not unidimensional
Maffulli et al. [46]	Italy (Italian)	NT				25	Inadequate	Cohen *k* = 0.78	Inadequate internal consistency statistic
Zwerver et al. [83]	Netherland (Dutch)	89	Adequate	PCA with VR on factors with eigenvalues > 1 74.6%	Three factors accounted together for 74.6 of the variance (First: items 2–6; Second: items 7–8; Third: item 1) (?)	83	Doubtful	Cronbach *α* = 0.73 (?)	Evidence that scale was not unidimensional
Hernandez-Sanchez et al. [26]	Spain (Spanish)	150	Very good	EFA with VR on factors with eigenvalues > 1 76.1%	Two factors accounted for 63.5% (items 1–6) and 12.6% (items 7–8) of variance, respectively (+)	150	Doubtful	Cronbach *α* = 0.885 (?)	Evidence that scale was not unidimensional
Lohrer et al. [42]	Germany (German)	NT				80	Doubtful	Cronbach *α* = 0.88 (?)	Unclear whether scale is unidimensional
Park et al. [60]	Korea (Korean)	NT				28	Doubtful	Cronbach *α* = 0.80 (?)	Unclear whether scale is unidimensional
Wageck et al. [81]	Brazil (Brazilian Portuguese)	NT				52	Doubtful	Cronbach *α* = 0.76 (?)	Unclear whether scale is unidimensional
Korakakis et al. [36]	Greece (Greek)	157	Very good	EFA with VR on factors with eigenvalues > 1 80.8%	One factor explained 80.8% of the variance, by excluding “the other knee injuries group” (+)	187	Very good	Cronbach *α* = 0.78 (?)	
Celebi et al. [7]	Turkey (Turkish)	89	Doubtful	CFA	1-factor solution tested *X*^2^ = NR, SRMR = NR, CFI = NR, RMSEA = NR, GFI = 0.88 (?)	89	Very good	Cronbach *α* = 0.78 (?)	
Kaux et al. [32]	Belgium (French)	NT				92	Doubtful	Cronbach α (0.88 to 0.90) (?)	Unclear whether scale is unidimensional
Hernandez-Sanchez et al. [25]	Spain (Spanish)	249	Very good	CFA	1-factor solution: *X*^2^ = 157.38 (*p* < 0.01), CFI = 0.80, GFI = 0.87, RMSEA = 0.17, SRMR = 0.10 (−) 2-factor solution: *X*^2^ = 67.94 (*p* < 0.01), CFI = 0.93, GFI = 0.94, RMSEA = 0.10, SRMSR = 0.06 (+)	249	Doubtful	Cronbach *α* = 0.74 (?)	Unclear whether scale is unidimensional. Alpha was not reported for 2-factor structure
Acharya et al. [1]	India (Kannada)	NT				35	Doubtful	Cronbach *α* = 0.99 (?)	Unclear whether scale is unidimensional
Pooled or summary result (overall rating)		785	Structure—inconsistent ( ±)			1086	Internal consistency—indeterminate (?)		Inconsistent results for structural validity which cannot be explained

*AT* Achilles tendinopathy, *CFA* confirmatory factor analysis, *CFI* comparative fit index, *EFA* exploratory factor analysis, *GFI* goodness of fit index, *NR* not reported, *NT* not tested, *PCA* principal component analysis, *RMSEA* root-mean-square error of approximation, *VR* varimax rotation, *SRMR* standardized root mean residuals, *SRMSR* standardized root-mean-square residual

**Fig. 2:**
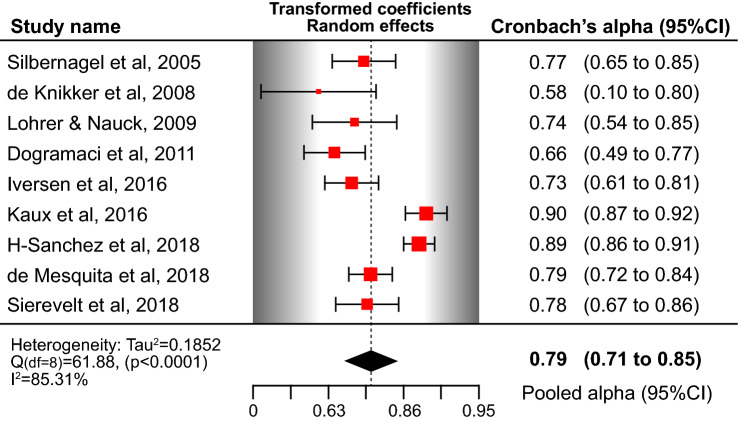
Forest plot of pooled Cronbach’s *α* coefficient for the Victorian Institute of Sport Assessment scale-Achilles (VISA-A). *CI* confidence intervals

Sufficient internal consistency of the unidimensional VISA-A was found (Table 3). The pooled Cronbach’s *α* using a random-effects model was 0.79 (Fig. 2). By subgrouping the studies that included only patients with Achilles tendinopathy or a mixed group of patients and asymptomatic individuals, the pooled estimate for alpha was 0.74 (95% CI 0.68–0.80) for patients and 0.87 (95% CI 0.82–0.92) for the mixed group. As the quality of evidence for internal consistency cannot be higher than the quality of evidence for structural validity [64], low-quality evidence suggests sufficient internal consistency for VISA-A.

#### VISA-G

Indeterminate evidence was found for VISA-G structural validity and internal consistency (criteria for “at least low evidence for sufficient structural validity” were not met [64]) (Tables 2, 3).

#### VISA-H

Low-quality evidence was found for insufficient unidimensionality of VISA-H and the results for internal consistency were ignored due to evidence of a 2-factor structure (Tables 2, 3).

#### VISA-P

Conflicting results were found on the structure of the VISA-P, this inconsistency could not be explained, and the evidence was not graded (Table 2).

Internal consistency received an indeterminate rating (?) due to inconsistent results for structural validity (Tables 2, 3) [64].

### Quality and results of studies evaluating cross-cultural validity/measurement invariance

No studies evaluated cross-cultural validity/measurement invariance of the VISA-A, VISA-G, or VISA-H. Only one study [25] of doubtful quality examined measurement invariance of VISA-P across sexes using multi-group confirmatory factor analysis. The difference of comparative fit index values was < 0.01 [9], indicating that VISA-P scores are comparable between men and women. However, examination of measurement invariance was performed in a model that did not met the requirements of sufficient unidimensionality [10, 64]. Low-quality evidence (very serious risk of bias) indicates sufficient measurement invariance between sexes for VISA-P.

### Internal structure in patients with other conditions

One study [43] of doubtful quality evaluated internal consistency of VISA-A in 39 patients with Haglund’s deformity providing indeterminate evidence as there is no information on the structural validity of the questionnaire in this population.

## Discussion

The most important finding of the present study was the very-low-quality evidence for the VISA questionnaires’ content and structural validity in assessing the severity of symptoms and disability in patients with lower limb tendinopathies.

More specifically, in relation to content validity, VISA-A presented very-low-quality sufficient relevance, insufficient comprehensiveness, and inconsistent comprehensibility. The VISA-G displayed moderate-quality evidence for sufficient comprehensibility and very-low-quality evidence of sufficient relevance and comprehensiveness. The VISA-P presented very-low-quality sufficient relevance, insufficient comprehensiveness, and inconsistent comprehensibility, while VISA-H presented very-low-quality evidence of insufficient content validity.

VISA-A displayed low-quality evidence for sufficient unidimensionality and internal structure, while for VISA-G, the rating was indeterminate. VISA-H presented low-quality evidence of insufficient unidimensionality. The structural validity of the VISA-P was inconsistent. Internal consistency for VISA-G, VISA-H, and VISA-P was indeterminate. Low-quality evidence from limited available data indicates sufficient measurement invariance between sexes for VISA-P.

### Content validity

The VISA questionnaires are routinely used as a core outcome measure in tendinopathy research and clinical practice [44, 52, 77, 78]. Content validity is the degree to which the content of an instrument is an adequate reflection of the construct to be measured [55]. The inadequate quality evidence supporting the content validity of the VISAs is unfortunately similar to other musculoskeletal questionnaires [10]. A lack of content validity affects all other measurement properties [74]. A recent consensus [65] recommended that at least content validity and internal structure should be adequate for recommending a scale as a core outcome set. This conflicts with the recent International Scientific Tendinopathy Symposium Consensus [78] that included the VISA questionnaires among the core domain set for tendinopathy. Several reasons may explain the inadequate evidence quality of VISA questionnaires: lack of or non-adherence to guidelines, lack of expertise in the research team, or poor reporting. Importantly, 40% of the included studies were conducted before the development and publication of the COSMIN guidelines in 2012. It is, therefore, not surprising that aspects of the included studies (development and cross-cultural adaptations) would not conform to these standards of the COSMIN initiative. Guidelines suggest patient input for good content coverage in PROM development studies [5, 61, 62], while content validity of existing PROMs can be assessed by asking patients about comprehensibility, comprehensiveness, and relevance of the items [74]. Patients are considered the “experts” in content validity assessment of PROMs [74]. Interestingly, in the present review, out of the 304 individuals recruited to evaluate content validity of the VISA questionnaires, the majority (*n* = 221) were asymptomatic individuals. Moreover, the majority of the studies assessed comprehensibility using diverse methodology, a few relevance, and none comprehensiveness—similar to a recent systematic review assessing content validity of PROMs for physical functioning in patients with low back pain [10].

Development and content validity of VISA-A, VISA-G, and VISA-H were modelled after VISA-P that was used as a background or structural framework. The content validity of VISA-A, VISA-H, and VISA-P questionnaires was established with limited inclusion of patients informally interviewed about their symptoms, by interviewing colleagues, using a focus group of clinicians with expertise in the area of tendinopathy. This results in a lack of meaningful patient-oriented qualitative exploration. Only the development of VISA-G included an adequate number of patients, but was limited to the assessment of comprehensibility. Mounting evidence suggests an association of psychological variables and outcome in tendinopathy, highlighting the need to address, from the patient’s perspective, the psychosocial factors in the evaluation of tendinopathy [48, 50, 63, 75]. Clinical research and empirical evidence have also underpinned other key features of tendinopathy that could plausibly be relevant for the construct of interest or replace existing items of the VISAs. For example, energy-storage and release activities, increases of the magnitude or rate of application of loading decline squat for patellar tendinopathy, or countermovement jump for Achilles tendinopathy are usually seen to increase symptoms in tendinopathy patients [44, 47, 68]. Important aspects of tendinopathy may be missing in the VISA questionnaires. It is suggested that the relevance as well as the comprehensiveness of the VISAs items require update and further investigation considering the current understanding of tendinopathy [49].

### Dimensionality and construct validity

Construct validity is the degree to which the scores of the PROM are consistent with predetermined hypotheses based on the assumption that the PROM validly measures the construct of interest [55]. In turn, structural validity is the degree to which the scores of a PROM are an adequate reflection of the dimensionality of the construct [55]. The VISA-A, VISA-H, and VISA-P questionnaires were formulated based on the hypotheses that the PROM will measure symptoms, function, and ability of patients to undertake sports as the domains of the same construct [6, 69, 80], while VISA-G was formulated to assess the severity of disability associated with greater trochanteric pain syndrome [20]. Interestingly, concerns were raised by this review as the VISA questionnaires do not share the same quality evidence for their underlying structure. Additionally, evidence suggests that the VISA questionnaires measure more than one construct and present violations of the assumptions of unidimensionality (Table 3). As such, a firm conclusion could not be reached with high-quality evidence regarding the underlying structure of the questionnaires. The 2-factor structure reported in development or cross-cultural adaptation studies (VISA-A, VISA-H, and VISA-P) included one or two items, mostly related with the physical activity section, suggesting that for the measurement of a broader, or a second construct important items maybe missing. From a different perspective, the scoring of the physical activity or sports participation section has been argued to substantially affect the scoring and consequently the underlying structure of the PROMs [49]. The VISA-A, VISA-H, and VISA-P were primarily designed for sporting populations; however, tendinopathy also occurs in non-sporting or sedentary individuals [14]. Item 8 (“sports”) of the VISAs is irrelevant to non-sporting/inactive individuals (e.g., greater trochanteric pain syndrome patients, sedentary individuals) [20, 67, 70]. This results in sedentary individuals scoring 0 in both items 7 and 8 and an underestimation of the total score irrespective of their symptoms. Inversely, the heavily weighted items 7 and 8 (40/100) in the overall scoring lead to an overestimation of the total score in high-level athletes that continue training with symptoms [49]. Moreover, the scoring formula of item 7 (0, 4, 7, or 10 points) has been argued to affect the variability of the scores, thus affecting the dimensionality analysis [25]. Modified versions of the VISA have been proposed or modifications of scoring of item 7 have been suggested to overcome this issue [25, 56, 67, 70].

According to COSMIN guidelines [54], evidence for structural validity is a prerequisite for the internal consistency and cross-cultural validity/measurement invariance of a PROM. Cronbach’s *α* can only be interpreted as a measure of internal consistency when the scale or subscale is unidimensional [12, 54]. Only the VISA-A displayed sufficient unidimensionality with acceptable internal consistency among its items; however, with low-quality evidence. These results were consistent with a recent reliability generalization meta-analysis of five studies reporting an alpha ranging from 0.70 to 0.79 [58]. Conversely, the internal consistency of the VISA-G, VISA-H, and VISA-P could not be rated due to inconsistent and indeterminate structural validity, and the lack of reporting of an alpha coefficient for each of the 2-factor structures, respectively. Given that when the assumption of unidimensionality is not met or evaluated the Cronbach’s *α* may overestimate the true internal consistency, pooled coefficients for VISA-P should be interpreted with extreme caution [59], and clinicians and researchers should be encouraged not to use the total scores of the PROM [54, 82].

Structural validity or measurement invariance requires that the items quantifying a construct of interest function in the same way across groups (e.g., between different cultures or genders) [23]. For example, significant gender differences in response to 12 weeks’ eccentric training in patients with Achilles tendinopathy have been documented [35]. It is currently not possible to determine if these are artefacts of the questionnaire or biological in nature [4]. It was confirmed by our review that measurement invariance is tested relatively infrequently in musculoskeletal research [23]. Low-quality evidence of measurement invariance was found, indicating that only VISA-P scores are comparable between Spanish men and women [25]. Future studies evaluating invariance of factor structure of VISA questionnaires across groups are much needed. Sparse information was available on the methodology and results of most included content validity studies. Future PROM development studies should: explain the item obtention and reduction method, prove pilot testing was conducted, and consult the COSMIN guidelines [74] on this measurement property. Finally, patient involvement in content validity studies is essential—the relevance and the comprehensiveness of the VISA questionnaires are yet to be adequately explored.

The validation process of the VISAs included only classical test theory methods, although approaches such as Rasch analysis have been advocated as more robust and useful in the evaluation of unidimensional PROMs [38]. Rasch analysis effectively evaluates the relevance and contribution of each item in measuring the underlying construct, the appropriateness of the response categories, and the amount of construct targeted by each item—overlooked properties in VISA validation studies. Rasch analysis should explore the unidimensionality of the VISA questionnaires. If it is violated, a refinement of the PROMs should be implemented by including the entire spectrum of the disease in their items [74].

A degree of subjectivity was necessary in the rating of the standards of the criteria of this newly formed guidelines, though the involvement of three reviewers and the pre-specified criteria helped to minimize the possibility of bias. Also, a weakness of our review was the consideration of different language versions of the PROMs as the same questionnaire in the evidence syntheses. However, this methodology has been recommended [74] and previously used [10]. Finally, COSMIN is an early set of guidelines with acknowledged limitations [64] that have to be evaluated in future research.

As suggested by patients and healthcare professionals from the International Scientific Tendinopathy Symposium Consensus, disability is among the nine domains of the core outcome set for tendinopathy. The VISA questionnaires have been recommended in research and clinical practice, because they are condition-specific composite scores of a mix of patient-rated pain and disability due to the pain, usually relating to tendon-specific activities [78]. Based on the COSMIN standards, none of the VISA questionnaires met the requirements to be rated as a category “A” PROM (recommended for use and the results obtained can be trusted) [64]. All VISA questionnaires were categorized as “B” PROMs, meaning that may have the potential to be recommended, but further validation studies are needed to assess their quality.

## Conclusion

Given the lack of alternative condition-specific outcome measures, we recommend the use of the VISAs in their current form, but the results obtained from their use should be interpreted with caution, especially for VISA-A, VISA-H, and VISA-P that presented insufficient or inconsistent ratings in content and structural validity. Researchers and clinicians should be using the VISA questionnaires in conjunction with other joint specific PROMs to capture the multifaceted presentation of the lower limb tendinopathies more adequately.

## Supplementary Information

Below is the link to the electronic supplementary material.

Supplementary file1 (DOCX 16 kb)

## Abbreviations

COSMIN: Consensus-based Standards for the selection of health Measurement Instruments
PROM: Patient-rated outcome measures
PRISMA: Preferred reporting items for systematic reviews and meta-analyses
VISA: Victorian Institute of Sport Assessment
VISA-A: Victorian Institute of Sport Assessment Achilles tendinopathy
VISA-G: Victorian Institute of Sport Assessment greater trochanteric pain syndrome
VISA-H: Victorian Institute of Sport Assessment proximal hamstring tendinopathy
VISA-P: Victorian Institute of Sport Assessment patellar tendinopathy
